# Effects of the Use of Air Purifier on Indoor Environment and Respiratory System among Healthy Adults

**DOI:** 10.3390/ijerph17103687

**Published:** 2020-05-23

**Authors:** Yoshiko Yoda, Kenji Tamura, Sho Adachi, Naruhito Otani, Shoji F. Nakayama, Masayuki Shima

**Affiliations:** 1 Department of Public Health, Hyogo College of Medicine, Nishinomiya 663-8501, Japan; naruhito@fa2.so-net.ne.jp (N.O.); shima-m@hyo-med.ac.jp (M.S.); 2 Center for Health and Environmental Risk Research, National Institute for Environmental Studies, Tsukuba 305-8506, Japan; ktamura@nies.go.jp (K.T.); fabre@nies.go.jp (S.F.N.); 3 Hyogo Regional Center of Japan Environment and Children’s Study, Hyogo College of Medicine, Nishinomiya 663-8501, Japan; sh-adachi@hyo-med.ac.jp

**Keywords:** air purifier, indoor environment, particulate matter, pulmonary function, fractional exhaled nitric oxide

## Abstract

Air purifiers have become popular among ordinary families. However, it remains controversial whether indoor air purification improves the respiratory health of healthy adults. A randomized crossover intervention study was conducted with 32 healthy individuals. The subjects were categorized into two groups. One group continuously used true air purifiers, and the other followed with sham air purifiers for 4 weeks. Following this first intervention, all the subjects underwent a 4-week washout period and continued with the second 4-week intervention with the alternate air purifiers. We collected fine particulate matter (PM) ≤ 2.5 µm in aerodynamic diameter (PM2.5), coarse particulate matter between 2.5 and 10 µm in aerodynamic diameter (PM10–2.5) and ozone (O_3_). The subjects’ pulmonary function and fractional exhaled nitric oxide (FeNO) were measured during the study period. The indoor PM2.5 concentrations decreased by 11% with the true air purifiers compared to those with sham air purifiers. However, this decrease was not significant (*p* = 0.08). The air purification did not significantly improve the pulmonary function of the study subjects. In contrast, an increase in the indoor PM10–2.5 and O_3_ concentration led to a significant decrease in the forced expiratory volume in one second (FEV_1.0_)/forced vital capacity (FVC) and maximal mid-expiratory flow (MMEF), respectively. In conclusion, air purification slightly improved the indoor PM2.5 concentrations in ordinary homes but had no demonstrable impact on improving health.

## 1. Introduction

Ambient air contains suspended particulate matter (PM) that varies in size [1,2]. Among this, fine PM ≤ 2.5 µm in aerodynamic diameter (PM2.5) is known to adversely affect the respiratory system when inhaled, inducing airway inflammation and other respiratory conditions [3,4,5,6]. Additionally, coarse PM with an aerodynamic diameter between 2.5 and 10 µm (PM10–2.5) also has adverse effects on human health [2]. Similarly, ozone (O_3_) is a gaseous air pollutant that can irritate the mucosal membranes of the eyes and respiratory system. Studies have also demonstrated that O_3_ exposure is associated with reduced pulmonary function [7,8,9] and asthma exacerbations [10]. Considering the adverse effects of PM and O_3_ on human health, reducing the ambient concentrations of these pollutants is desirable. Furthermore, as people spend the majority of their time indoors [11], lowering the indoor concentrations of air pollutants is crucial. Air purification is considered to be effective in reducing indoor PM concentrations [12,13,14,15,16].

In countries outside Japan, air purification has been reported to reduce the indoor concentrations of PM_2.5_ and allergens, alleviating the symptoms of people with asthma [17,18,19,20,21]. Moreover, air purification has been shown to improve cardiovascular and pulmonary functions in elderly individuals [22]. However, there are also many contradictory reports showing no demonstrable changes in cardiovascular and pulmonary functions in elderly individuals [23,24]. As can be seen from these studies, the effects of air purification have been evaluated in elderly individuals or in patients with respiratory diseases (such as asthma). To date, only a small number of studies have examined the effects in healthy individuals, and these studies were performed exclusively in countries with relatively high PM concentrations [25,26]. Thus, there have been very few research efforts that have assessed the effects of air purification in the context of everyday life in healthy individuals living in developed countries with low PM concentrations, such as Japan. In ordinary homes, indoor PM concentrations are affected not only by outdoor PM concentrations [27] but also by domestic emission sources (including cooking on a gas stove) and the activities of household members (such as opening and closing windows) [28,29,30]. Therefore, changes in indoor PM concentrations associated with the use of air purification systems must be evaluated under everyday life conditions and not in a special experimental environment. Additionally, certain air purifiers are known to generate O_3_ [31]. Since O_3_ can potentially be harmful to the respiratory system, its indoor concentrations should also be assessed.

Indoor air pollutants could adversely affect human health no matter how low their concentrations are. This is because the duration of exposure to these pollutants increases during the time people live their normal day-to-day lives indoors. Thus, in the present study, a crossover intervention study was conducted in healthy adults during their usual everyday activities to elucidate the association between an improved indoor environment achieved by air purification and its potential health effects. For this purpose, we measured the concentrations of PM and O_3_ in ordinary homes and repeatedly performed the measurements of pulmonary function on the study subjects.

## 2. Materials and Methods 

### 2.1. Study Design

**Figure 1:**
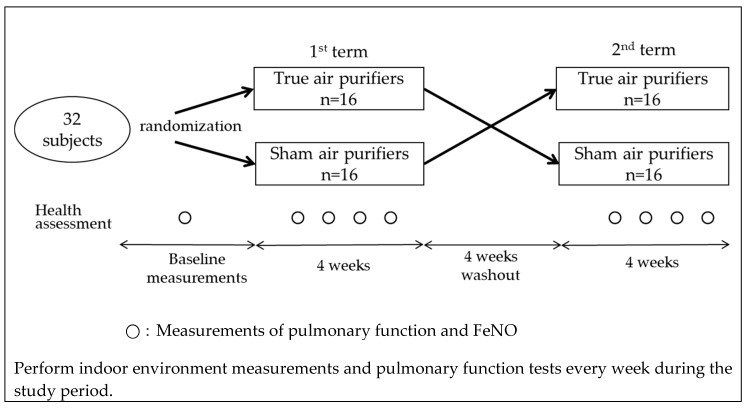
Study flowchart.

A crossover intervention study was conducted between November 2018 and February 2019 in the Hanshin area in the urban districts of Osaka and Hyogo prefectures in Western Japan. We recruited individuals who lived alone or did not have any smokers in their households and were living in homes without any air purifiers at the time of recruitment. A total of 32 healthy non-smoking adult volunteers (aged 30–60 years) agreed to participate in the study. The subjects were randomly assigned to one of two groups (16 each). Each subject in the first group received a true air purifier equipped with filters, while members of the second group were given sham air purifiers that had no filters. All the subjects maintained their usual lifestyles for 4 weeks. Following this first intervention, they underwent a 4-week washout period and then continued with a second 4-week intervention using the alternate air purifier (Figure 1). To eliminate potential data variability introduced by differences in air purifiers, all the subjects used the same model (EP-NZ30, Hitachi, Tokyo, Japan). This air purifier is equipped with a dust collection and deodorization filter and can catch more than 99% of particles with a diameter between 0.1 and 2.5 µm. Owing to the design of this air purifier, the presence or absence of filters cannot be determined based on its appearance. Over the course of the present study, the air purifier was placed in the living room of each subject’s home and was continuously used in a fixed flow mode.

At the beginning of the study, the subjects were evaluated using a questionnaire that included the following questions: the number of household members, presence or absence of pets, living environment, medical history of asthma, and presence or absence of rhinitis. During the study, the subjects were also surveyed every week with a different questionnaire containing questions related to the ventilation conditions of their homes, routine living activities, and respiratory symptoms.

The present study was approved by the Ethics Review Board of Hyogo College of Medicine (Registered No. 2898) and performed in accordance with the Declaration of Helsinki, after obtaining written informed consent from each participating subject.

### 2.2. Sample and Data Collection

During the course of the study, we measured the concentrations of PM and O_3_ both indoors and outdoors.

Samples of airborne PM were captured on filters using an impactor (ATPS-20H; Sibata Scientific Technology, Soka, Japan) that could fractionate PM into fine particles (PM2.5) and coarse particles (PM10–2.5). The impactor was connected to a small vacuum pump (MP-∑300N IIT; Sibata Scientific Technology, Soka, Japan) adjusted to 1.5 L/min; the pump was operated intermittently (5 min on followed by 30 min off) and the filters were collected once every week. In each subject’s home, one vacuum pump was placed in the living room for indoor sampling and another pump was installed on the balcony for outdoor sampling. The sampling of PM was conducted indoors and outdoors in a similar way. The mass concentrations of PM2.5 and PM10–2.5 were measured by weighing the filters before and after sampling using an electronic microbalance with a sensitivity of 0.1 µg (UMX-2, Mettler-Toledo Inc., Columbus, OH, USA) after storage for more than 24 h at a constant temperature (21.5 ± 0.2 °C) and relative humidity (35 ± 5%).

The O_3_ was collected using a passive sampler (Ogawa & Co., Ltd., Kobe, Japan) [32]. The sampler uses a filter coated with a nitrite-based solution which is oxidized to nitrate by O_3_. The sampler was placed continuously at the same locations as the PM-capturing pumps and changed to new ones every week. After air sampling, the samplers were stored at 4 °C until the analyses. The concentrations of O_3_ were analyzed according to Tang et al. [33]. In brief, the filter was placed in an extract vial and 5 mL of ultra-pure water (Milli-Q) was poured in the vial. After shaking gently, the supernatant solution was analyzed by ion chromatography. The detection limit of this assay was 0.1 µg/mL, which corresponded to 0.2 ppb for a one-week average of O_3_.

The indoor air temperature and humidity were determined using a HOBO data logger (Onset Computer Corp., Bourne, MA, USA). The outdoor air temperature and humidity data were downloaded from a nearby monitoring station.

Pulmonary function testing was performed using a spirometer (Microspiro HI-205T. Chest, M.I., Inc., Tokyo, Japan) and the following parameters were evaluated: forced vital capacity (FVC), forced expiratory volume in one second (FEV_1.0_), forced expiratory volume in one second (FEV_1.0_)/forced vital capacity (FVC), maximal mid-expiratory flow (MMEF), peak expiratory flow rate (PEF), and the ratio of the maximum expiratory flow rate at 50% of the FVC to the maximum expiratory flow rate at 25% of the FVC ($\overset{\cdot }{V}$_50_/$\overset{\cdot }{V}$_25_). Prior to each examination, the spirometer was calibrated to 3 L using a syringe. The airway inflammation was assessed by measuring the fractional exhaled nitric oxide (FeNO) with NObreath (Bedfont Scientific, Maidstone, UK). The device was calibrated prior to use, and the ambient NO concentration was measured every day as a background level. FeNO can be measured in the range of 10–30 °C by this device, and the detection limit is 1 ppb. The study subjects visited our laboratory once per week to receive pulmonary function and FeNO measurements. These measurements were performed according to the American Thoracic Society guidelines [34,35,36].

### 2.3. Statistical Analyses

The arithmetic mean and standard deviation were calculated for each of the indoor air pollutant levels and the parameters of pulmonary function. The PM and O_3_ were detected and quantified for all samples. As the FeNO measurement data were not normally distributed, a logarithmic transformation of the data was performed before the statistical analysis.

The weekly indoor concentrations of air pollutants with a true air purifier were compared with those in the rooms with a sham air purifier. Indoor PM concentrations are dependent on outdoor PM concentrations. Therefore, weekly indoor-to-outdoor concentration ratios (I/O ratios) of PM [27] were used to assess the associations between the effect of air purification and living environment factors. We used a t-test to evaluate a hypothesis.

When analyzing the association between the effect of air purification and the results of pulmonary function testing, a mixed model which estimates both fixed and random effects was employed to allow for repeated measurements of the function. We controlled for air temperature; humidity; and subjects’ body mass index, age, and sex as fixed-effect covariates. The true and sham air purifiers were coded with dummy variables and treated as a fixed effect. The results were expressed as percentage change with a 95% confidence interval (CI).

The relationships between the indoor concentrations of air pollutants and the parameters of pulmonary function testing were analyzed using a mixed effect model after adjusting for air temperature, humidity, and the subjects’ body mass index. For each pollutant, the results were presented as changes in the parameters of pulmonary function and FeNO per interquartile range (IQR) increase in pollutant level (which was defined as the mean of the concentrations obtained during the 7-day period before each pulmonary function testing).

The level of statistical significance was set at *p* < 0.05. All the statistical analyses were performed using SPSS 22 (IBM Co., Armonk, NY, USA).

## 3. Results

### 3.1. Descriptive Statistics 

There were no dropouts and all the subjects successfully completed the study. Table 1 shows the baseline characteristics of the study subjects. They consisted of 10 men (mean age 41.8 ± 8.9 years) and 22 women (mean age 40.9 ± 6.9 years). Most parameters of pulmonary function were larger in the males than in the females. However, the $\overset{\cdot }{V}$_50_/$\overset{\cdot }{V}$_25_ was lower and the FeNO was higher than in males than in females. These gender differences were considered to be due to the high prevalence of history of allergic diseases among the male subjects (data not shown).

The mean value for the PM2.5 concentrations reported during the study period by the nearby air quality monitoring station was 14.9 ± 4.2 µg/m^3^. Table 2 shows the results of an indoor air quality measurement performed during the study period. The mean values of the indoor PM2.5 concentrations were 8.6 ± 5.0 µg/m^3^ for homes with a true air purifier and 9.7 ± 4.3 µg/m^3^ for homes with a sham air purifier. Thus, the use of a true air purifier led to a decrease in indoor PM2.5 concentration by approximately 11%. However, this decrease was not statistically significant (*p* = 0.08). Similarly, the use of a true air purifier slightly reduced both the PM10–2.5 and O_3_ concentrations, but with no statistical significance.

The I/O ratio of PM2.5 was lower in homes with a true air purifier (0.72 ± 0.38) than those with a sham air purifier (0.81 ± 0.53). However, this difference was not significant (*p* = 0.11). Additionally, there were no significant differences in the I/O ratios of PM10–2.5 and O_3_.

### 3.2. Indoor Air Pollutant Levels in Relation to Living Environment Factors 

**Figure 2:**
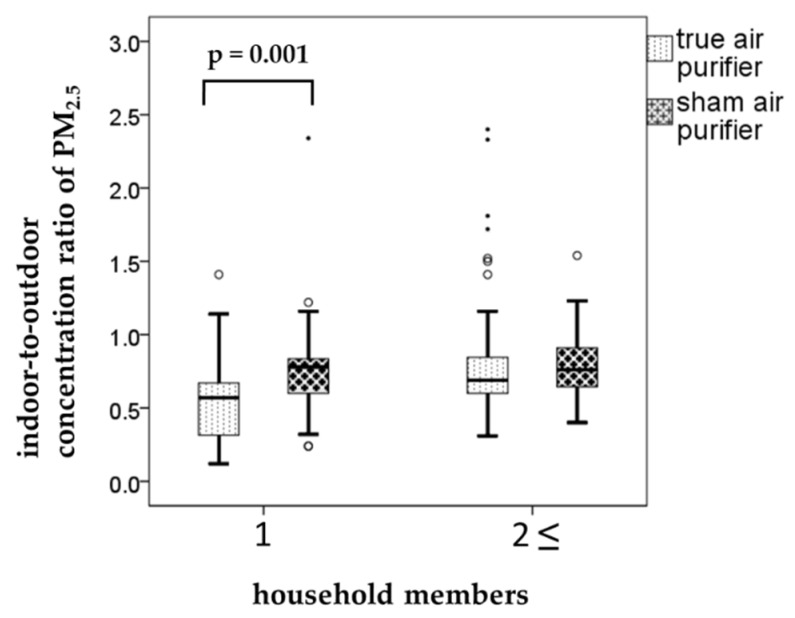
The relationships between the particulate matter (PM) ≤ 2.5 µm in aerodynamic diameter (PM2.5) concentrations and household members. Boxplots shows weekly indoor-to-outdoor concentration ratio (I/O ratio) by household members when using the true and sham air purifiers. Each box shows the median, 75th, and 25th percentiles. The top and bottom of the whisker indicate the maximum and minimum. The mall circles are outliers. The points are extreme outliers.

When we assessed the relationships between the air pollutant levels and living environment factors, the effect of air purification was evident in single-person households. In these households (n = 10), the weekly I/O ratios of PM2.5 were 0.56 ± 0.28 and 0.76 ± 0.35 for the true and sham air purifiers, respectively (*p* = 0.001, Figure 2). This clearly demonstrates the effectiveness of air purification in reducing air pollutant levels. Air purification also lowered the I/O ratio of PM2.5 in multi-person households (n = 22). However, the decrease was marginal and statistically insignificant. Other factors associated with living environment examined in the present study were the presence or absence of pets and flooring materials (tatami (thick woven straw mat), wood, or carpet). The results of these examinations indicated that the effect of air purification (as measured by differences in the I/O ratio of PM2.5) was not significantly related to these factors.

### 3.3. Associations Air Pollutant Levels and Pulmonary Function 

Using a mixed model, we next analyzed the relationship between the use of a true or sham air purifier and the subjects’ pulmonary function testing data. The results are shown in Table 3. There was a trend for the FEV_1_, FEV_1.0_/FVC, MMEF, PEF, and FeNO to be slightly lower in subjects who used a true air purifier compared with those who used a sham air purifier. However, none of these differences reached statistical significance.

Table 4 shows changes in the parameters of pulmonary function testing per IQR increase in the indoor concentrations of air pollutant (PM2.5, PM10–2.5, or O_3_). An increase in the indoor PM10–2.5 concentration led to a significant decrease in the FEV_1.0_/FVC (−0.52% (95% CI: −1.00, −0.05) for an IQR increase of 1.86 µg/m^3^). Similarly, an increase in the indoor O_3_ concentration resulted in a significant decrease in the MMEF (−0.23 L/s (95% CI: −0.40, −0.07) for an IQR increase of 2.81 ppb). In contrast, the FVC significantly increased when the indoor concentrations of PM2.5 and PM10–2.5 rose. However, the changes were modest.

## 4. Discussion

The present study was a crossover intervention study designed to elucidate the association between the reduction in indoor concentrations of air pollutants (achieved by air purification) and its potential health effects in ordinary homes in the context of everyday life. The results indicated that indoor PM2.5 concentrations decreased in homes with a true air purifier as compared to those with a sham air purifier. This decrease was statistically significant in a subgroup analysis including only single-person households. However, the overall analysis showed no significant difference. Similarly, the air purification was not significantly associated with the pulmonary function of the study subjects.

Air purifiers are recognized to be effective in reducing indoor PM concentrations. In a study performed in Shanghai, China, air purification led to a reduction in the indoor PM2.5 concentration by as much as approximately 57% (from 96.2 to 41.3 µg/m^3^) when the rooms used for the study were kept closed for 48 h [26]. In the present study, air purification also resulted in a slight decrease in the indoor PM2.5 concentration under everyday life conditions, but the decrease was statistically insignificant. Ambient PM2.5 concentrations in Japan have shown a declining trend every year. During this study period, the mean outdoor PM2.5 concentration was 12.5 ± 3.83 µg/m^3^. We speculate that air purification failed to reduce the PM2.5 concentrations significantly due to the naturally low concentrations of this air pollutant in the present study. A similar argument could be used to explain the results obtained with PM10–2.5.

Changes in indoor PM concentrations are reportedly affected by the environment of household members [37]. Sood et al. reported that emission reductions achieved by upgrading household cookstoves would lead to an improved indoor environment [38]. We also investigated the effects of the household environment on the changes in indoor air pollutant levels. The results showed that air purification was able to lower the pollutant levels in single-person households. In these homes, there was little foot traffic and doors were kept closed for extended periods of time. Furthermore, single-person households had lower rates of indoor PM emissions. These factors would account for the observed effect of air purification.

Solar irradiation contributes to the secondary formation of O_3_. Thus, ambient O_3_ concentrations tend to be higher during spring and summer. As the present study was conducted in winter, the O_3_ concentrations were low both indoors and outdoors and especially low indoors. Certain air purifiers are known to generate O_3_ [31]. However, the air purifier used in this study uses a filter system and does not generate O_3_. Therefore, there was very little difference in O_3_ concentration between homes with a true air purifier and those with a sham air purifier.

The results presented here indicated that there was no significant reduction in indoor air pollutant levels by air purification, and changes in the study subjects’ pulmonary function were not observed. Previous reports showed that air purification decreased indoor PM2.5 concentrations and contributed to improved pulmonary function [18,25]. However, studies conducted in elderly individuals reported no association between air purification and improved pulmonary function [23,24]. Thus, there is still no clear consensus regarding the effect of air purification on pulmonary function. A recent investigation demonstrated that air purification lowered PM2.5 concentration by 72.4%, resulting in decreased airway inflammation [39]. However, the same investigation failed to detect a significant improvement in pulmonary function (FEV_1.0_ and FVC). Similarly, when the effectiveness of air cleaning devices was evaluated at home in adult patients with asthma, Pedroletti et al. found no significant improvement in pulmonary function, although the FeNO values decreased significantly [40]. Hence, while air purification can certainly improve indoor environments, its impact on airway inflammation and pulmonary function remains controversial because the results vary depending on, for example, the study location and subject characteristics.

The results of the present study did not show that air purification can improve pulmonary function or reduce airway inflammation. However, an increase in indoor PM10–2.5 concentration resulted in a significant decrease in FEV_1.0_/FVC. Similarly, an increase in the indoor O_3_ concentration led to a significant decrease in MMEF, even though the concentrations were considerably low. These results suggest that increased concentrations of PM10–2.5 and O_3_ may cause an obstructive impairment of pulmonary function. In our previous study, significant decreases in FEV_1.0_ among healthy adolescents were observed in relation to increases in PM10–2.5 concentrations [41]. On the other hand, in the present study increased indoor concentrations of PM2.5 and PM10–2.5 led to a slight but significant increase in FVC. Weichenthal et al. reported that a decrease in the PM2.5 concentration achieved by air purification was associated with statistically significant improvements in FEV_1.0_ and PEF [25]. Park et al. examined the effects of air purification in children with asthma and/or allergic rhinitis, and the results demonstrated that the use of air purifiers reduced indoor PM2.5 concentrations and improved the evening PEF [18]. These results are not entirely consistent with our present findings. However, they underscore the need for further studies to determine how air purification influences pulmonary function by improving indoor environments.

One limitation in the present study was the relatively small subject sample size (32 households). Preferably, these clinical studies should be performed with a larger number of participants. However, in practice only a limited number of subjects can be recruited to perform trials under strictly controlled conditions after installing air purifiers in each subject’s home. Another limitation was that windows were not always fully closed in each subject’s room where the present investigation was conducted. This hampered the precise evaluation of the effectiveness of air purification. Pacitto et al. suggested the possibility that the I/O ratios of certain pollutants can be considerably reduced when air purifiers were used in rooms with windows kept closed [42]. However, windows are frequently opened and closed under everyday life conditions. In this regard, our study design that did not restrict subjects’ activities (such as opening and closing windows) was ideal for assessing the effects of air purification in the context of everyday life. Lastly, the study subjects did not always stay in the rooms used for pollution monitoring throughout the entire day. This precluded the accurate assessment of the effects on the subjects’ pulmonary function by the changes in indoor pollutant levels that resulted from air purification.

The strength of the present study is that it recruited only healthy individuals and not patients with respiratory disorders. This allows for the generalization of our findings. Based on the information presented here, if indoor environments improve by air purification, it is expected to contribute to formulating strategies for preventing health problems in the future.

## 5. Conclusions

In the present study, air purification slightly improved indoor environments in ordinary homes but without reaching statistical significance. Additionally, we were not able to clearly show the effect of air purification on health improvement. However, we successfully demonstrated that air purification can reduce indoor PM2.5 concentrations in single-person households relative to outdoors. Future studies should further investigate, with larger populations, how long-term air purification helps improve human health in the context of everyday life.

## Figures and Tables

**Table 1:** Baseline characteristics of the study subjects (mean ± SD).

	Male (*n* = 10)	Female (*n* = 22)	Total (*n* = 32)
Age (years)	41.8 ± 8.9	40.9 ± 6.9	41.2 ± 7.5
Height (cm)	169.0 ± 6.0	158.8 ± 4.3	162.0 ± 6.8
Weight (kg)	66.5 ± 13.3	53.3 ± 6.1	57.4 ± 10.7
BMI (kg/m^2^)	23.2 ± 3.9	21.2 ± 2.7	21.8 ± 3.2
FVC (L)	3.87 ± 0.44	2.94 ± 0.32	3.23 ± 0.56
FEV_1.0_ (L)	3.16 ± 0.35	2.43 ± 0.26	2.66 ± 0.45
FEV_1.0_/ FVC (%)	82.1 ± 7.04	82.8 ± 6.23	82.6 ± 6.39
MMEF (L/s)	3.31 ± 1.00	2.83 ± 0.83	2.98 ± 0.90
PEF (L/s)	8.19 ± 1.83	5.29 ± 0.99	6.20 ± 1.87
$\overset{\cdot }{V}$_50_/$\overset{\cdot }{V}$_25_	2.97 ± 0.87	3.59 ± 1.24	3.40 ± 1.16
FeNO (ppb) *	23.9 ± 2.1	10.8 ± 1.9	13.9 ± 2.2

SD, standard deviation; BMI, body mass index. * Geometric mean ± geometric standard deviation.

**Table 2:** Measurement results of each concentration using true and sham air purifiers during the study period (mean ± SD).

	1st Term			2nd Term			Total		
	True Air Purifiers	Sham Air Purifiers	*p* Value	True Air Purifiers	Sham Air Purifiers	*p* Value	True Air Purifiers	Sham Air Purifiers	*p* Value
**Indoor**									
PM2.5 (µg/m^3^)	8.6 ± 3.8	9.7 ± 4.4	0.170	8.6 ± 5.9	9.7 ± 4.3	0.270	8.6 ± 5.0	9.7 ± 4.3	0.085
PM10–2.5 (µg/m^3^)	3.1 ± 2.1	3.4 ± 2.4	0.500	2.1 ± 1.3	2.3 ± 1.9	0.531	2.6 ± 1.8	2.8 ± 2.2	0.355
O_3_ (ppb)	1.6 ± 1.5	2.5 ± 2.0	0.002	2.8 ± 2.3	2.2 ± 1.4	0.079	2.2 ± 2.0	2.4 ± 1.7	0.446
Temp (°C)	18.7 ± 1.7	18.7 ± 2.4	0.984	16.6 ± 2.4	17.7 ± 2.3	0.020	17.7 ± 2.3	18.2 ± 2.4	0.088
RH (%)	53.1 ± 7.6	50.2 ± 8.4	0.048	47.0 ± 10.8	46.0 ± 9.0	0.554	50.1 ± 9.7	48.2 ± 8.9	0.109
**Outdoor**									
PM2.5 (µg/m^3^)	12.8 ± 5.0	12.9 ± 5.1	0.972	12.2 ± 1.8	12.1 ± 1.5	0.789	12.5 ± 3.8	12.5 ± 3.8	0.973
PM10–2.5 (µg/m^3^)	8.9 ± 7.6	9.1 ± 7.8	0.906	6.2 ± 1.8	6.1 ± 1.4	0.672	7.6 ± 5.6	7.6 ± 5.9	0.938
O_3_ (ppb)	20.9 ± 5.6	23.1 ± 6.5	0.045	29.3 ± 5.4	27.0 ± 5.7	0.025	25.1 ± 6.9	25.0 ± 6.4	0.892
**I/O** **ratio**									
PM2.5	0.73 ± 0.28	0.84 ± 0.69	0.123	0.71 ± 0.47	0.78 ± 0.29	0.263	0.72 ± 0.38	0.81 ± 0.53	0.110
PM10–2.5	0.51 ± 0.43	0.59 ± 1.00	0.262	0.36 ± 0.27	0.38 ± 0.34	0.074	0.43 ± 0.36	0.49 ± 0.76	0.182
O_3_	0.08 ± 0.08	0.12 ± 0.11	0.470	0.11 ± 0.10	0.08 ± 0.06	0.071	0.09 ± 0.09	0.11 ± 0.09	0.052

Temp, temperature; RH, relative humidity; I/O ratio, indoor-to-outdoor concentration ratio.

**Table 3:** Estimated percent changes in the pulmonary function test results comparing true and sham air purifiers.

	Percent Changes (95% CI)	*p* Value
FVC (L)	0.001 (−0.062, 0.065)	0.966
FEV_1_ (L)	−0.008 (−0.065, 0.050)	0.797
FEV_1.0_/ FVC (%)	−0.322 (−1.662, 1.018)	0.637
MMEF (L/s)	−0.071 (−0.219, 0.078)	0.352
PEF (L/s)	−0.135 (−0.490, 0.220)	0.455
$\overset{\cdot }{V}$_50_/$\overset{\cdot }{V}$_25_	0.239 (−0.278, 0.756)	0.362
Log_FeNO	−0.001 (−0.097, 0.095)	0.978

CI, confidence interval.

**Table 4:** Effects of an increase in the interquartile range of each indoor pollutant on the parameters of pulmonary function.

	Indoor PM2.5		Indoor PM10–2.5		Indoor O_3_	
	Percent Changes (95% CI)	*p* Value	Percent Changes (95% CI)	*p* Value	Percent Changes (95% CI)	*p* Value
FVC (L)	0.02 (0.00, 0.04)	0.020	0.02 (0.00, 0.05)	0.035	−0.02 (−0.09, 0.05)	0.660
FEV_1_ (L)	0.01 (−0.01, 0.03)	0.315	0.01 (−0.01, 0.03)	0.554	−0.06 (−0.12, 0.00)	0.058
FEV_1.0_/FVC (%)	−0.35 (−0.77, 0.08)	0.108	−0.52 (−1.00, −0.05)	0.030	−1.41 (−2.88, 0.06)	0.061
MMEF (L/s)	−0.01 (−0.06, 0.04)	0.723	−0.02 (−0.08, 0.03)	0.406	−0.23 (−0.40, −0.07)	0.005
PEF (L/s)	0.07 (−0.05, 0.19)	0.228	0.10 (−0.03, 0.23)	0.137	−0.31 (−0.69, 0.08)	0.117
$\overset{\cdot }{V}$_50_/$\overset{\cdot }{V}$_25_	0.13 (−0.10, 0.37)	0.273	0.17 (−0.08, 0.42)	0.174	−0.31 (−0.80, 0.19)	0.225
Log_FeNO	0.03 (−0.01, 0.06)	0.123	−0.01 (−0.04, 0.03)	0.708	−0.09 (−0.19, 0.01)	0.083

The interquartile range of PM2.5, PM10–2.5, and O_3_ are 4.09 µg/m^3^, 1.86 µg/m^3^, and 2.81 ppb respectively.

## Abbreviations

PM	particulate matter
PM2.5	fine particulate matter ≤ 2.5 µm in aerodynamic diameter
PM10–2.5	coarse particulate matter between 2.5 and 10 µm in aerodynamic diameter
O_3_	ozone
FEV_1.0_	forced expiratory volume in one second
FVC	forced vital capacity
MMEF	maximal mid-expiratory flow
PEF	peak expiratory flow rate
$\overset{\cdot }{V}$_50_/$\overset{\cdot }{V}$_25_	The ratio of the maximum expiratory flow rate at 50% of the FVC to the maximum expiratory flow rate at 25% of the FVC
FeNO	fractional exhaled nitric oxide
I/O ratio	indoor-to-outdoor concentration ratio
SD	standard deviation
BMI	body mass index
CI	confidence interval
